# Clinical, Prognostic, and Biological Features of High‐Risk Cardiometabolic Phenotype: The REMODEL Study

**DOI:** 10.1002/mco2.70631

**Published:** 2026-02-26

**Authors:** Adam A. R. Muhammad, Jia Kai Chuan, Aisyah Latib, Jennifer A. Bryant, Vivian Lee, Redha Boubertakh, Thu‐Thao Le, Calvin W. L. Chin

**Affiliations:** ^1^ National Heart Research Institute Singapore National Heart Centre Singapore Singapore Singapore; ^2^ Cardiovascular Sciences Academic Clinical Program Duke NUS Medical School Singapore Singapore

1

Traditional risk stratification in cardiometabolic diseases relies on markers such as blood pressure (BP), glucose, and lipid levels. While informative, these parameters fail to capture the full biology underpinning cardiometabolic risk. Notably, nearly 30% of hypertension‐related health burdens occur in patients with apparently well‐controlled BP [1], highlighting the limitations of conventional metrics and the need to uncover mechanistic pathways driving vulnerability. In this study, our objectives are to identify distinct risk profiles using the KAMILA algorithm [2], and characterize the clinical, prognostic, and biological features that define high‐risk hypertensive phenotype.

The **REMODEL** (Response of the myocardium to hypertrophic conditions in the adult population; clinicaltrials.gov unique identifier: NCT02670031) is a prospective, observational cohort of asymptomatic individuals with essential hypertension [3]. Exclusion criteria included secondary causes of hypertension, known cardiovascular disease, inherited cardiomyopathies, atrial fibrillation, or contraindications to gadolinium or CMR. All participants underwent 24 ambulatory BP monitoring (OnTrak 90227, SpaceLabs Healthcare) and standardized cardiovascular magnetic resonance (CMR; see supporting information for details), with de‐identified images centrally analyzed at the **National Heart Research Institute of Singapore CMR Core Laboratory** using standardized protocols [4].

Measurement of 192 serum proteins related to cardiovascular diseases were performed using 2 commercially available multiplex immunoassays (Olink Target Cardiovascular Disease II and III, Olink Proteomics, Uppsala, Sweden). This proteomic analysis enabled us to investigate molecular pathways that may contribute to adverse outcomes observed in high‐risk hypertensive phenotype (see online supporting information). The primary outcome was a composite of first occurrence hypertension‐related adverse events: acute coronary syndromes, first heart failure hospitalization, strokes and all‐cause mortality. Patients were followed until December 2024, with data censored at the last known event‐free date for individuals lost to follow‐up. Clinical events were adjudicated through medical record review using predefined criteria previously published [3]. The cause of death was ascertained from the National Death Registry.

Continuous variables were summarized as mean ± SD or median (IQR) where appropriate, and categorical variables as counts and percentages. Between‐group comparisons were performed using *t*‐tests or Mann‐Whitney *U* tests for continuous data, and χ^2^ tests for categorical data. Event‐free survival was assessed with Kaplan–Meier analysis with log‐rank testing. All analyses were performed in R (v4.4.1) and Python (v3.12.4), with a two‐sided *p* < 0.05 considered as statistically significant.

Of the 885 individuals with hypertension, 21% had diabetes mellitus and 49% had dyslipidemia. The mean body mass index was 26.4±4.5 kg/m^2^ and 24‐h BP was 131±14/79±10 mmHg. Among the 83 candidate predictor variables assessed, four were selected for their superior discriminative performance: N‐terminal pro‐Brain Natriuretic Peptide (NT‐proBNP), Romhilt‐Estes electrocardiographic score, and indexed interstitial and myocyte volumes—both derived from cardiovascular magnetic resonance (CMR). This combination yielded an area under the receiver operating characteristic curve (AUC) of 0.76 (95% CI: 0.68–0.83; *p*<0.001) for predicting adverse outcomes. In contrast, the best‐performing clinical predictor, 24‐h systolic BP, ranked eighth with an AUC of 0.66 (95% CI: 0.57–0.74; *p*<0.001). The silhouette method identified two optimal clusters, with the highest silhouette score of 0.76. Unsupervised clustering of the cohort was performed using the KAMILA algorithm, and the two clusters demonstrated clear separation in principal component analysis (see online supporting information and Figure 1 in medRvix doi: https://doi.org/10.64898/2026.01.16.26344303 for details). Predictor selection was outcome‐informed to enrich for clinically meaningful phenotypes, while cluster assignment itself was performed without outcome labels. This hybrid approach was designed to enhance clinical interpretability but may inflate associations between cluster membership and outcomes; and should therefore be considered hypothesis‐generating.

These clusters included a low‐risk (*n* = 787) and a high‐risk (*n* = 98) group. Individuals in the high‐risk group were more likely males, younger (54±14 vs. 58±10 years, *p*<0.001), worse renal function and a greater visceral adiposity (200.0 [148.1, 297.3] vs. 160.7 [103.1, 232.0] cm^2^, *p* = 0.007) and higher 24‐h blood pressures (144±16/84±13 vs. 129±13/79±9 mmHg). Individuals in the high‐risk cluster also had more adverse features of cardiac remodeling on both CMR and circulating biomarkers (Figure 1A). Of note, individuals in both clusters had similar subcutaneous adiposity (*p* = 0.291). During a follow‐up period of 60 [37, 73] months, the high‐risk cluster experienced significantly worse outcomes (Figure 1B; *p*<0.001); and remained significant even after adjusting for potential confounders (HR 8.89 [95% CI 4.75–16.65]; *p* < 0.001).

**FIGURE 1:**
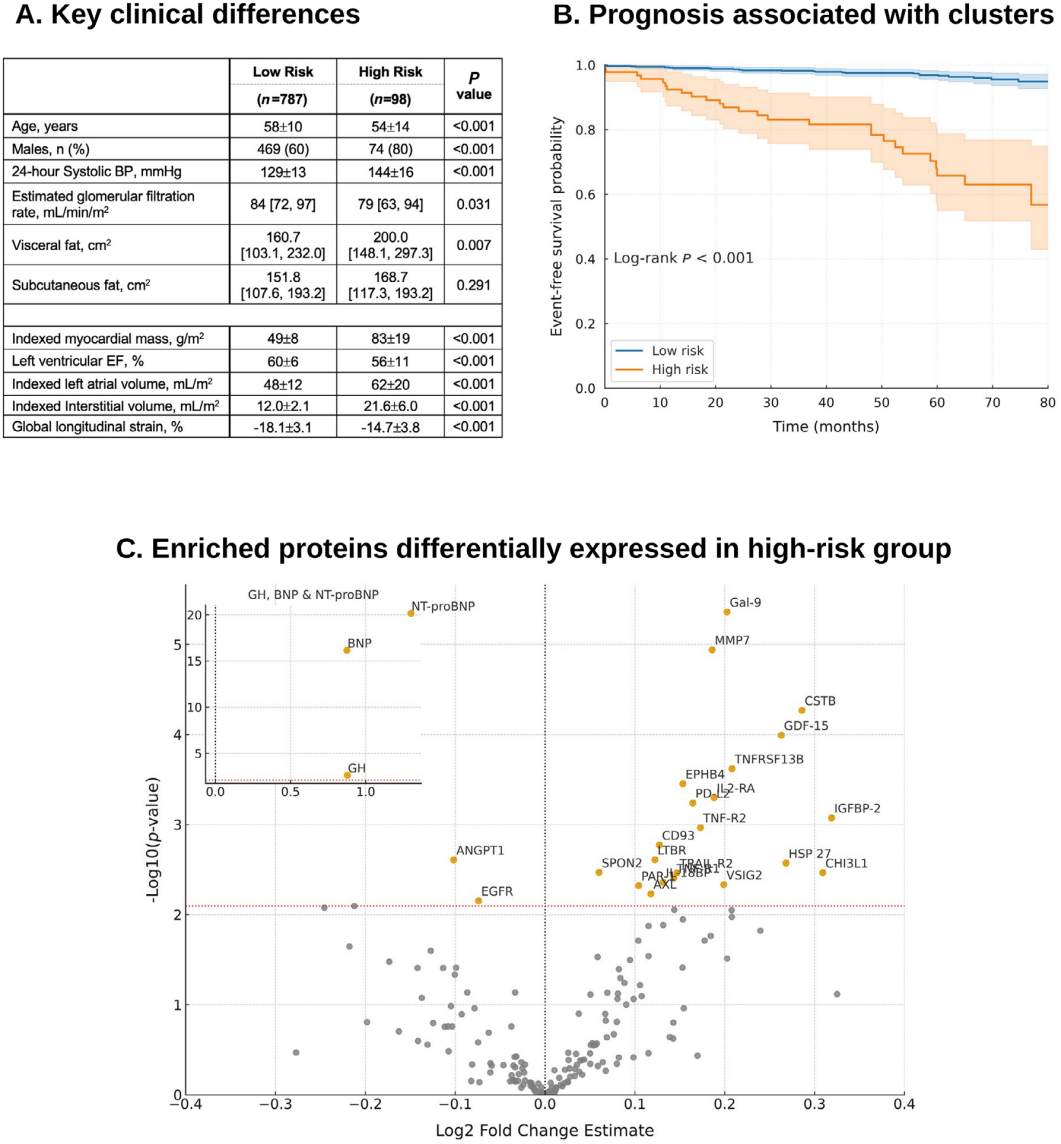
Defining clinical, prognostic, and biological features associated with high‐risk cluster. Those in the high‐risk cluster were younger, more likely males, greater visceral adiposity, had worse renal function and higher 24‐h blood pressures (Panel A). High‐risk cluster was also associated with significantly worse prognosis (Panel B). Abbreviations: BP blood pressure; EF ejection fraction. Proteomic profiling identified 26 circulating proteins significantly enriched in the high‐risk cluster (Panel C).

Proteomic profiling revealed 26 circulating proteins significantly enriched in the high‐risk hypertensive cohort (Figure 1C). Classical cardiac stress biomarkers (BNP and NT‐proBNP) were prominently elevated, consistent with myocardial stretch and neurohormonal activation. Proteins mediating extracellular matrix turnover and fibrosis (MMP7, GDF‐15, CSTB, and HSP27) were also significantly enriched. A broad set of immune regulators and cytokine mediators (Galectin‐9, TNF‐R1, TNF‐R1, TRAIL‐R2, TNFRSF13B, IL2‐RA, IL‐18BP, PD‐L2, CD93, CHI3L1, LTBR, and VSIG2) suggested heightened inflammatory activity, immune checkpoint modulation, and pro‐apoptotic signaling. In parallel, vascular signaling and angiogenic proteins (EPHB4, ANGPT1, AXL, EGFR, PAR‐1, and SPON2) implicated endothelial dysfunction, angiogenesis, and thrombo‐inflammatory processes.

The identification of the four key predictors underscores the importance of integrating multimodal data, including biomarkers and advanced imaging, for risk assessment. NT‐proBNP, a well‐established marker of cardiac stress, has been consistently linked to adverse cardiovascular outcomes. Beyond this, proteomic profiling provided additional mechanistic insights into the high‐risk hypertensive phenotypes. Twenty‐six proteins were significantly enriched, spanning pathways of myocardial stretch, extracellular matrix remodeling, inflammation, apoptosis, and vascular dysfunction. Taken together, these circulating signatures complement structural insights from CMR‐derived measures of interstitial and myocyte volumes, which reflect microstructure and subclinical remodeling; and electrocardiographic findings, such as the Romhilt‐Estes score which capture left ventricular hypertrophy [5]. Proteomic enrichment of natriuretic peptides therefore reflects internal biological coherence rather than independent validation of cluster distinctiveness.

Visceral adiposity emerged as a key clinical feature of the high‐risk cluster. Despite similar levels of subcutaneous fat, individuals in this group exhibited substantially greater visceral fat burden, a phenotype strongly associated with systemic inflammation, insulin resistance, and adverse cardiometabolic outcomes. Importantly, the high‐risk cluster was paradoxically younger, suggesting that adverse cardiovascular risk in hypertension may be driven by early‐onset metabolic and myocardial vulnerability rather than chronological aging alone. This finding highlights limitations of conventional risk stratification tools, which often underestimate lifetime risk in younger individuals with adverse biological profiles.

This study is novel in demonstrating that unsupervised risk clustering not only improves prediction but also reveals disease biology, linking clinical features with molecular signatures to refine risk assessment. However, it has several limitations. The modest high‐risk sample limits generalizability. Although two clusters were optimal by silhouette score, finer sub‐phenotypes may emerge in larger cohorts. Use of all‐cause mortality broadened capture of hypertension‐related fatal outcomes but reduced mechanistic specificity compared with cardiovascular mortality alone. The model showed moderate discrimination, and comparison with Framingham or SCORE was not feasible due to missing key variables. Future studies should validate these findings in larger, multiethnic cohorts; and incorporate mediation and sensitivity analyses to clarify pathways associating cluster membership to outcomes.

## Author Contributions

TTL and CWLC contribution to the conception and design of the study. AARM, JKC JAB, VL and RB contributed to the acquisition, analysis, and interpretation of the data. AARM and JKC drafted the manuscript under the supervision of TTL and CWLC. All authors made critical revisions to the manuscript, gave final approval and agreed to be accountable for all aspects of work ensuring integrity and accuracy.

## Funding Information

The study is funded by the National Medical Research Council of Singapore (MOH‐CSAINV23jan‐0001; NMRC/CG1/003/2021‐NHCS and MOH‐OFLCG22may002).

## Ethics Approval Statement

Ethics approval was obtained from the local centralized institutional review board (2015/2603), and all participants provided written informed consent. ClinicalTrials.gov identifier: NCT02670031.

## Conflicts of Interest

The authors declare no conflicts of interest.

## Supporting information




**Supporting File 1**: mco270631‐sup‐0001‐SupMat.docx

## Data Availability

Data available on request from the corresponding author.
